# Case report of ascending colon cancer and multiple jejunal GISTs in a patient with neurofibromatosis type 1 (NF1)

**DOI:** 10.1186/s12885-019-6375-9

**Published:** 2019-12-05

**Authors:** Liang Shang, Zhen Fang, Jin Liu, Fengying Du, Haiyan Jing, Yali Xu, Kangdi Dong, Xiaoman Zhang, Hao Wu, Changqing Jing, Leping Li

**Affiliations:** 1 Department of Gastroenterological Surgery, Shandong Provincial Hospital Affiliated to Shandong University, Jinan, Shandong China; 2 Department of Gastroenterology, Shandong Provincial Hospital Affiliated to Shandong University, Jinan, Shandong China; 3 Department of Pathology, Shandong Provincial Hospital Affiliated to Shandong University, Jinan, Shandong China; 4 Department of ENT, Shandong Provincial ENT Hospital, Shandong Provincial ENT Hospital Affiliated to Shandong University, Jinan, Shandong China; 5 Department of Gastrointestinal Surgery, Shandong Provincial Hospital Affiliated to Shandong University, Jingwuweiqi Street, 324, Jinan, 250021 Shandong China

**Keywords:** NF1, GIST, Colon cancer

## Abstract

**Background:**

NF1(Neurofibromatosis type 1) is an autosomal dominant genetic disorder. Patients with NF1 have an increased risk of developing benign or malignant tumours, such as gastrointestinal stromal tumours (GISTs). However, the coexistence of NF1, GIST and colon cancer is very rare, and few cases have been reported in the literature.

**Case presentation:**

We admitted a case of a 64-year-old man with type 1 neurofibromatosis, GISTs, and ascending colon cancer. This case was characterized by café-au-lait macules, discrete cutaneous neurofibromas, nodular neurofibromas, multiple jejunal tumours, and ascending colon cancer. Laparoscopic exploration revealed ascending colon cancer and multiple jejunal tumours. Laparoscopic right hemicolectomy and local excision of the jejunal tumours were performed successfully. The pathological results confirmed moderate differentiated adenocarcinoma of the ascending colon with multiple jejunal GISTs (low risk, very low risk). Moreover, the immunohistochemistry results of multiple jejunal GISTs suggest that NF1 is positive. Whole-exome sequencing (WES) of colon cancer revealed mutations in more than 20 genes, including *KRAS, PIK3CA, APC, SMAD4,* etc. The results of whole-exome sequencing (WES) of jejunal GISTs revealed an *NF1* mutation and no KIT or PDGFR gene mutation.

**Conclusion:**

We report a rare case of simultaneous NF1, GIST and colon adenocarcinoma. For patients with NF1, benign and/or malignant tumours are often combined. Therefore, these patients should undergo regular physical examinations so that early detection and early treatment can be achieved.

## Background

NF1 is an autosomal dominant disease with an incidence of approximately 1 in 2600 to 3000 individuals. Approximately one-half of the cases are inherited [1]. NF1 affects multiple organ systems and has a wide range of variable clinical manifestations [2]. The most characteristic clinical manifestations of NF1 are café-au-lait macules and multiple neurofibromas. Other manifestations include neurological symptoms, skeletal dysplasias and visceral injuries. The pathogenic gene for NF1 is located on the autosome 17q11.2. The gene encoding NF1 was defined in 1991 [3]. In the case of the disease, this chromosomal locus is deleted so that the patient cannot produce the corresponding neurofibromin protein. Neurofibromatosis protein is a tumour suppressor that slows cell proliferation by accelerating the reduction of the proto-oncogene p21-ras, which plays a major role in the intracellular mitosis signal transduction system. Its function and role in tumourigenesis and other manifestations of NF1 have been studied in depth.

Patients with NF1 have an increased risk of developing GISTs and other benign or malignant tumours [4–6]. In patients with NF1, GIST often occurs in the small intestine (more than 70%), usually with multiple tumours that have different molecular pathologies [7, 8].

Currently, there is no definitive treatment, and clinical management is limited to monitoring and symptomatic treatment for specific complications, usually surgery.

## Case presentation

**Fig. 1:**
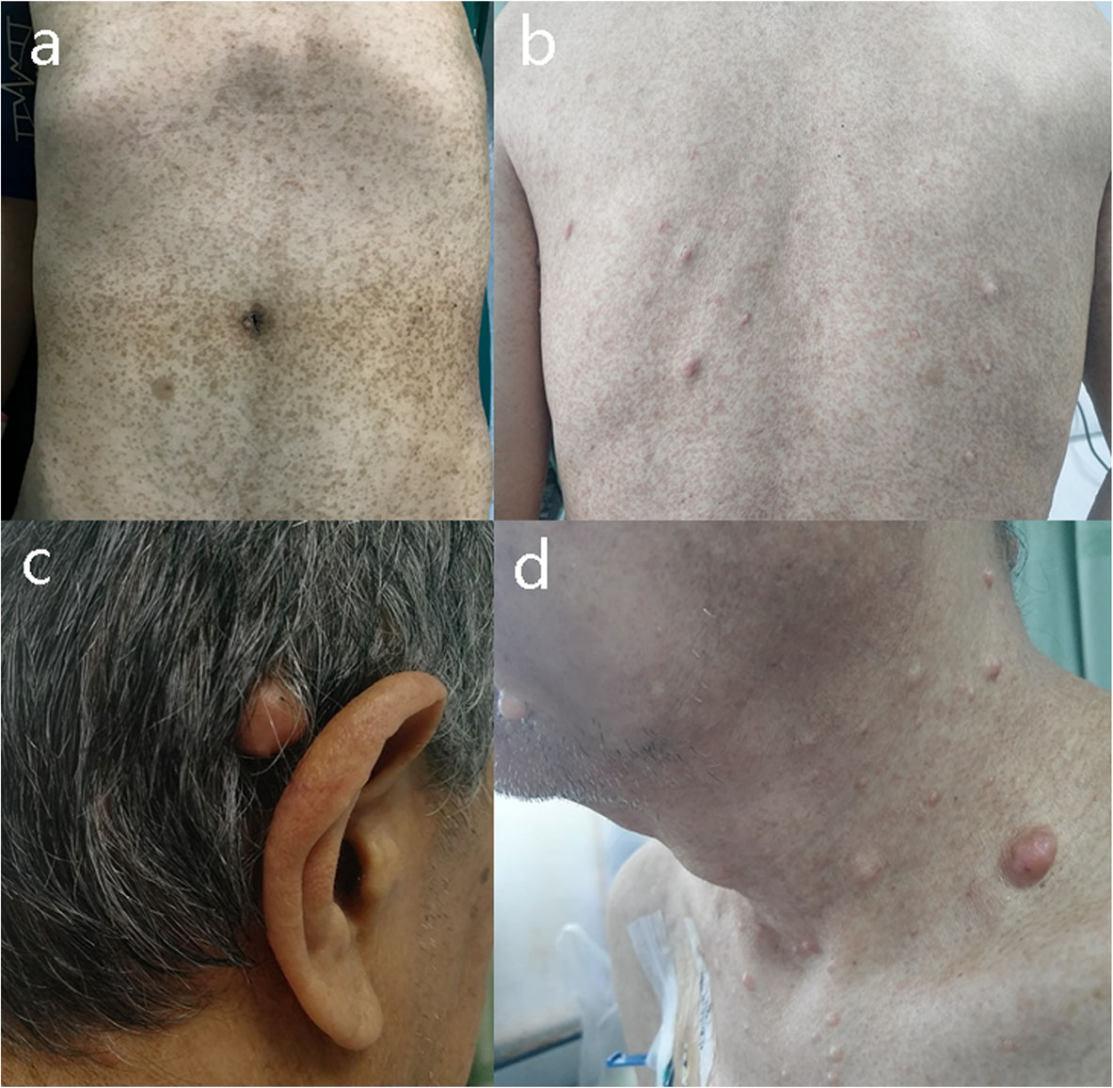
**a**, **b** Café-au-lait macules, **c**, **d** Neurofibromas

**Fig. 2:**
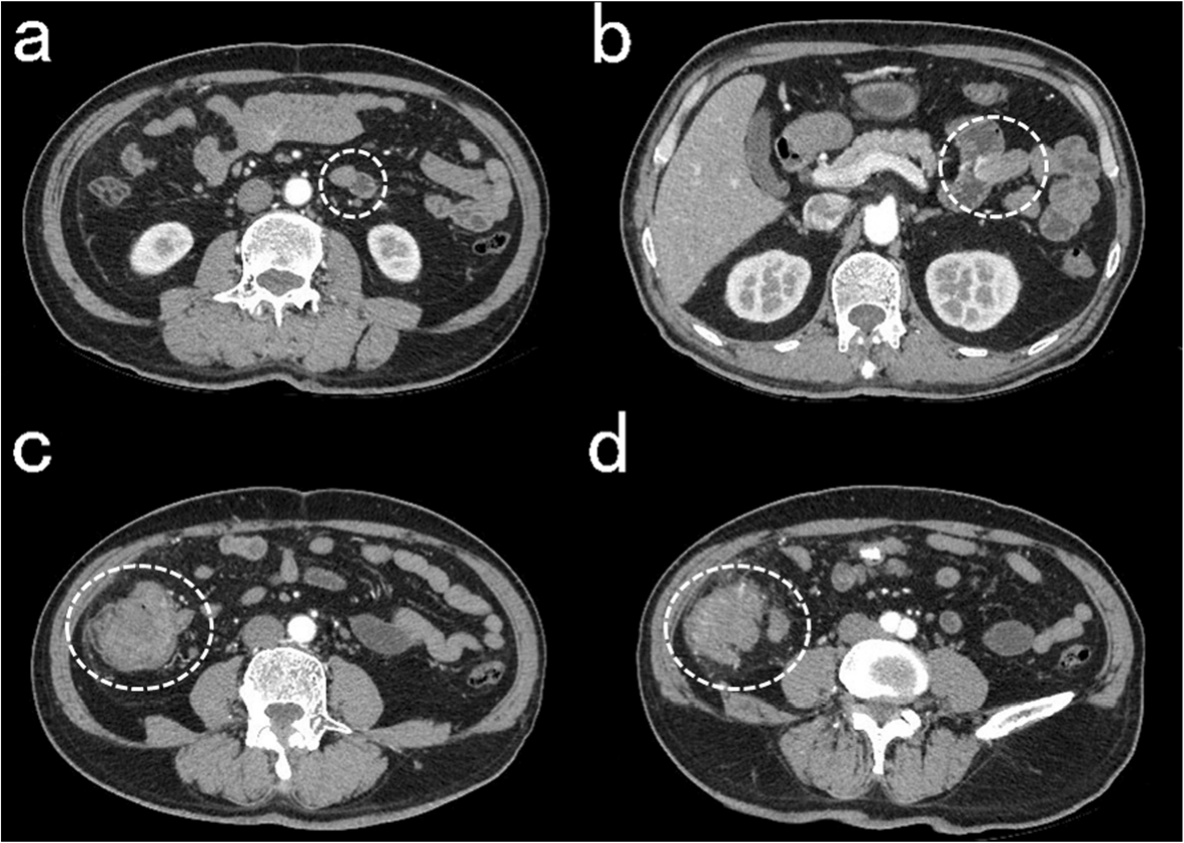
CT (**a**, **b)** Jejunal GISTs, **c**, **d** Ascending colon cancer

A 64-year-old man was admitted to our hospital on September 2, 2018 because of abdominal pain with low fever for more than 2 months. The patient had a history of NF1. At the age of 14 years, subcutaneous nodules began to appear in the elbow and gradually began to appear throughout the whole body. The patient had no history of hypertension, coronary heart disease, diabetes, etc. Physical examination revealed café-au-lait macules of varying sizes and multiple subcutaneous nodules and skin fibromas. (Fig. 1) Colonic endoscopy revealed that the mass was located in the ascending colon near the ileocaecum, and the surface of the tumour was erosive. Pathological examination of a colon tumour biopsy revealed moderate differentiated adenocarcinoma. Blood tests showed mild anaemia (HB 101 g/L), and tumour markers and coagulation indicators were normal. Abdominal contrast-enhanced computed tomography (CT) scan showed irregular thickening of the ascending colon walls and inhomogeneous enhancement, consistent with colon cancer CT performance. Multiple enlarged lymph nodes were observed around the tumour. CT also revealed multiple jejunal masses, and the clinical impression may be GISTs (Fig. 2). The clinical diagnoses were ascending colon cancer (cT4aN + M0), NF1, and multiple jejunal tumours (GISTs?). None of his family had a similar performance.

**Fig. 3:**
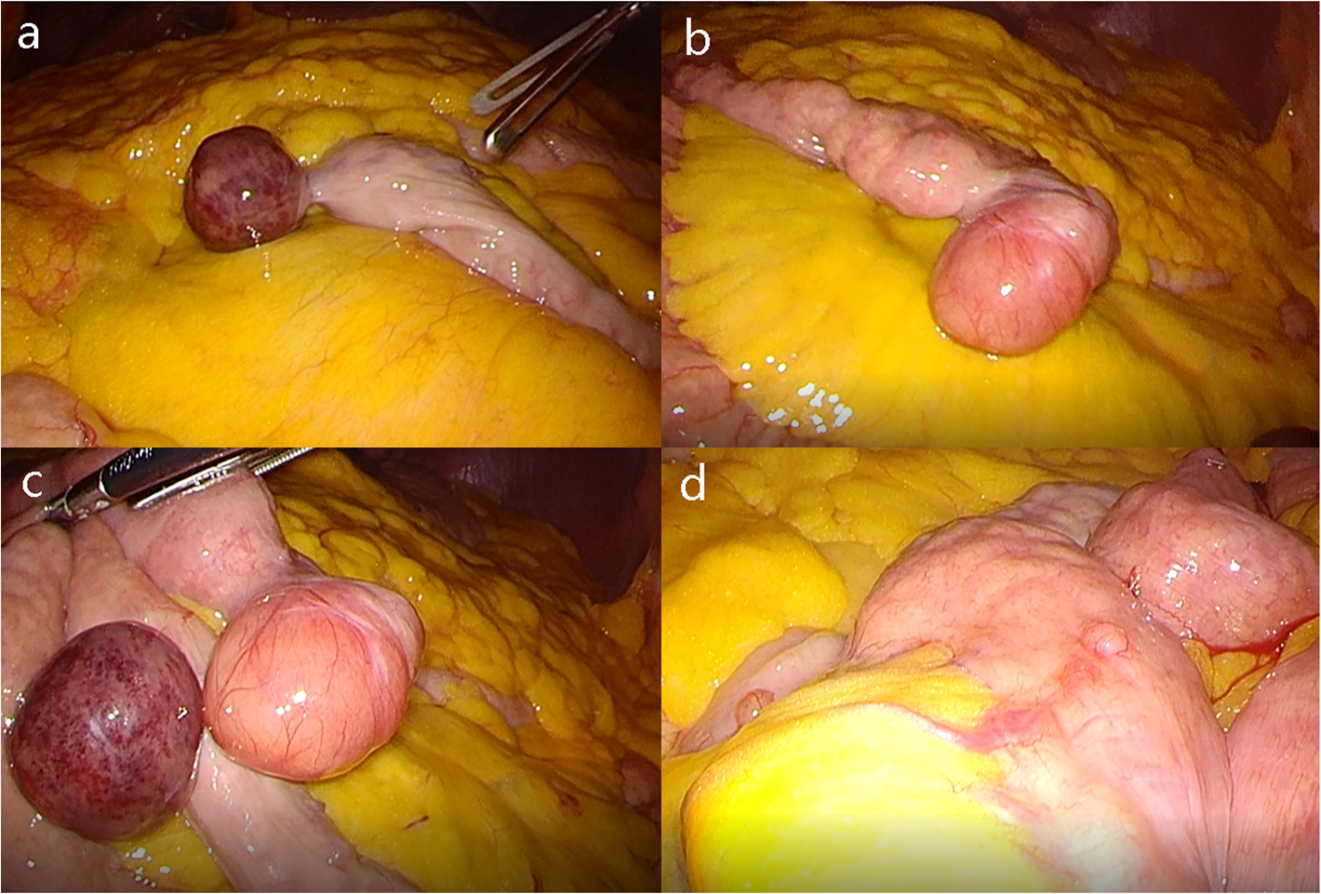
Laparoscopic exploration showing jejunal tumours (**a**, **b**, **c**, **d**)

During laparoscopic exploration, a tumour was found in the ascending colon, which manifestly invaded the serous membrane, and multiple enlarged lymph nodes were observed around the tumour. Additionally, multiple small intestine masses were observed in the jejunum within the range of 10–50 cm from Treitz’s ligament. The largest mass was approximately 3 cm, and the smaller masses were approximately 0.5–2 cm in diameter. (Fig. 3) Subsequently, the ascending colon and jejunal tumours were excised under laparoscopy.

Pathology and whole exon sequencing were performed on the ascending colon and multiple jejunal tumours.

Pathologically, the resected ascending colon tumour was a moderately differentiated adenocarcinoma with one lymph node metastasis (1/18) – pT4a pN1a cM0 stage IIIB according to the TNM staging of AJCC Guidelines Version 3.2018.

**Fig. 4:**
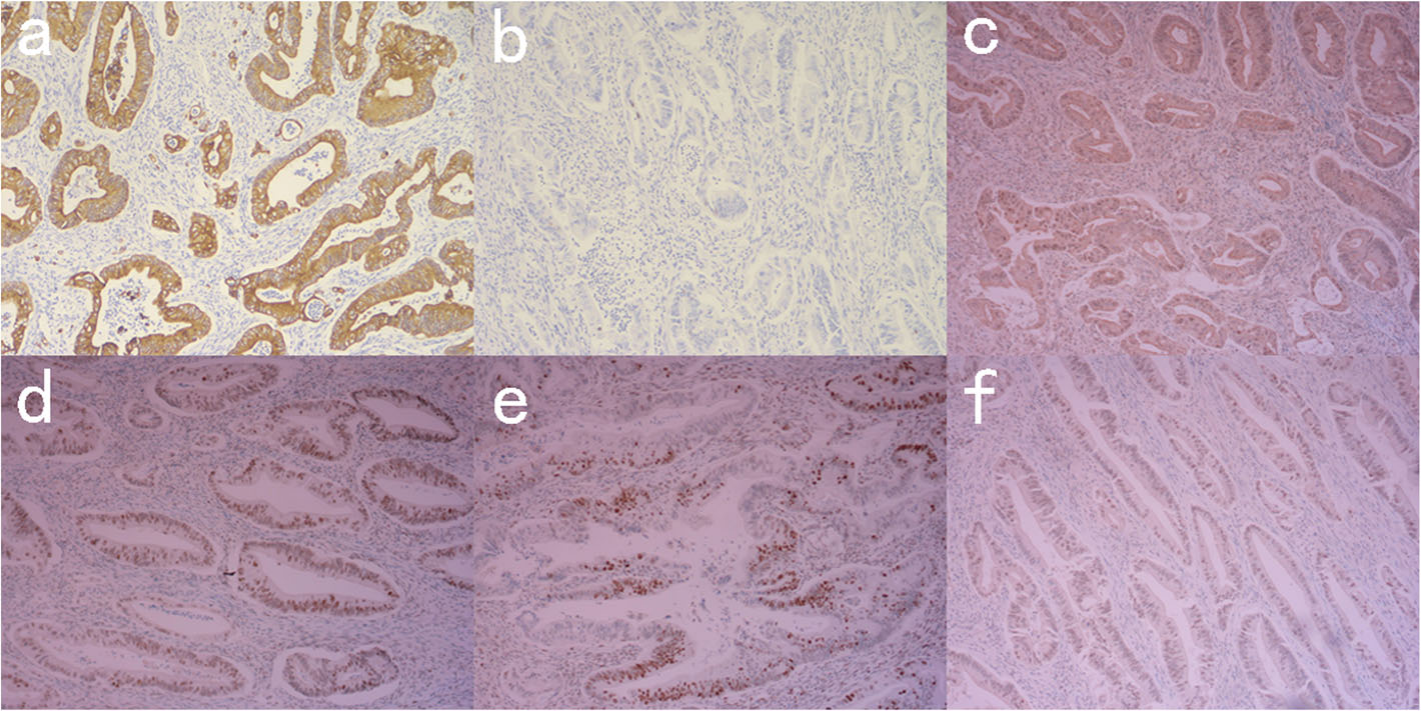
IHC of ascending colon cancer (**a**) CK818+, **b** wt-p53, **c** MLH1+, **d** MSH2+, **e** MSH6+, **f** PMS2+

**Fig. 5:**
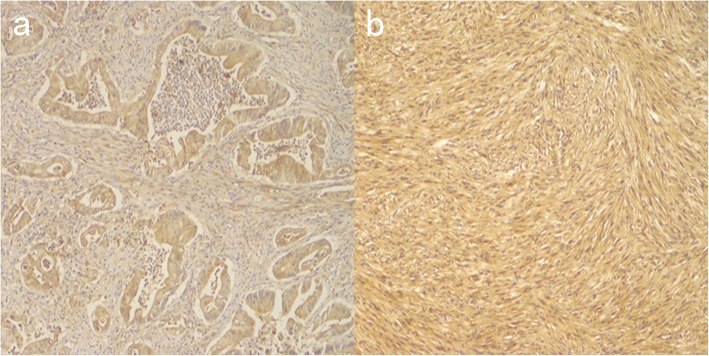
IHC of NF1 (**a**) Ascending colon cancer, **b** Jejunal GISTs

**Fig. 6:**
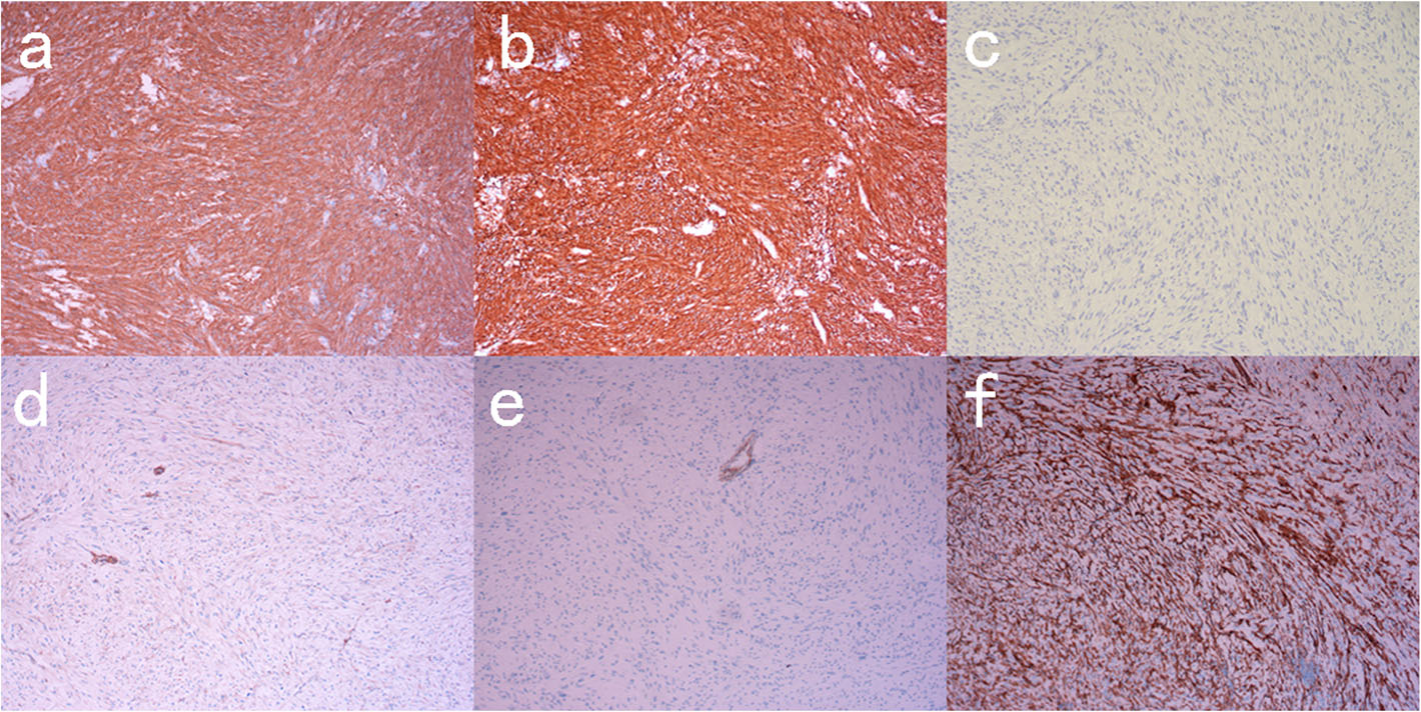
IHC of jejunal GISTs (**a**) CD117+, **b** dog-1+, **c** CK818-, **d** SMA+, **e** desmin-, **f** CD34+

The IHC results for colon cancer: wt-p53, CK818+, MLH1+, PMS2+, MSH2+, MSH6+. (Fig. 4) Furthermore, immunohistochemistry results suggest that NF1 is over-stained. (Fig. 5a) The pathological result for jejunal tumours indicated low or very low risk GIST (< 5/50 HPF; 3.7 cm, 2.5 cm, 0.5 cm), positive for CD117, Dog-1, CD34, SMA, negative for desmin, CK818. (Fig. 6) Immunohistochemistry results indicated that NF1 was positive. (Fig. 5b).

The whole-exome sequencing (WES) results of colon adenocarcinoma revealed mutations in 29 genes, including *KRAS, PIK3CA, APC, SMAD4,* etc. The WES results for stromal tumours showed an NF1 mutation, and no PDGFRA or KIT mutation. The specific characteristics of colon cancer and GISTs are shown in Table 1.

**Table 1:** Specific characteristics of colon adenocarcinoma and GISTs

	Colon adenocarcinoma	GIST (1)	GIST (2)	GIST (others)
Location	Ascending colon	10–50 cm from the Treitz’s ligament	10–50 cm from the Treitz’s ligament	10–50 cm from the Treitz’s ligament
Size (cm)	5 × 3 × 3	3.7 × 2.2 × 2.8	2.5 × 2.5 × 2.3	0.5 × 0.5 × 0.6
Grade	moderately differentiated	low risk	low risk	very low risk
IHC	NF1 pan-stained, wt-p53, CK818+	NF1+, CD117+, Dog-1+, CD34+, S-100-, E-cad-, CK818-, SMA+, Caldesmon+, β-catenin+, E-cad-, Ki-67+ (4%)	NF1+, CD117+, Dog-1+, CD34+, S-100-, E-cad-, CK818-, SMA+, desmin-	CD117+, Dog-1+, S-100-, SMA+, desmin-
	MLH1+, PMS2+, MSH2+, MSH6+			
WES	*KRAS, PIK3CA, APC, SMAD4*	*NF1, MSS,* TMB (low)	*NF1, MSS,* TMB (low)	–
	*APOB, ATPAF2, CACNA1H, COL6A3, DECR2, DLG2, DOK3, DYNC1I1, HCN1, KIAA1841, KIF6, KLRG1, MYOD1, NEFH, OR10C1, OR8A1, PTPN7, RGS18, RHOBTB1, SPATS2L, STAC, SYNPO2L, TAS1R2, USH2A, VEPH1*			

*GIST* Gastrointestinal stromal tumours, *NF1* Neurofibromatosis type 1, *IHC* Immunohistochemistry, *WES* Whole-exome sequencing, *MSS* Microsatellite stability, *TMB* Tumour mutation load, *wt-p53* Wild-type p53

The patient recovered smoothly and was discharged on the 7th day after surgery. Because the risk level of jejunal GISTs was low and very low, and imatinib was not administered after surgery. We continued follow up. To date, the patients have undergone 12 cycles of chemotherapy (FOLFOX regimen). No recurrence was observed in the 12-month follow-up period.

## Discussion and conclusions

Neurofibromatosis type 1 (NF1), also known as von Recklinghausen disease, is caused by a mutation in the *NF1* gene located on chromosome 17q11.2 [9, 10]. Neurofibromin is a protein product encoded by *NF1* that is expressed in many tissues, including the spleen, kidneys, brain, and thymus.

NF1 has a variety of diagnostic features, such as multiple neurofibromas, freckles, café-au-lait macules and iris nodules [11]. If GIST is present in patients with NF1, the GIST usually does not have a *KIT* mutation [12, 13]. Therefore, the response of patients with NF1-related GISTs to tyrosine kinase inhibitor imatinib treatment is poor [13]. In this case, the patient had multiple intestinal stromal tumours, and WES revealed *NF1* mutations and no *KIT* or *PDGFRA* mutations. This finding indicates that the formation of GISTs may be related to an *NF1* mutation.

Patients with NF1 are more likely to develop malignant tumours, such as breast cancer, colorectal cancer, stomach cancers, etc. [14] Eui Tae Kim reported 125 cases of NF1 patients in 2012, among which malignancy occurred in 16 patients. The location of the tumour includes the central nervous system, lung, breast, stomach, small intestine, colon, liver, etc. [15] Patients with NF1 are more likely than normal people to develop malignant tumours.

Studies have reported that neurofibromin can directly inhibit the activation of the RAS signalling pathway. The inactivation of NF1 primarily leads to the uncontrolled activation of RAS [16]. Uncontrolled RAS activation induces the activation of mitogen-activated protein kinase (MAPK) and extracellular signal-regulated kinases 1 and 2 (ERK1 and ERK2). The activation of RAF/MAPK stimulates transcription and cell growth. The activation of the aberrant RAS signalling pathway also leads to cell proliferation and survival through other pathways, such as the PI3K-mTOR pathway [17]. Whole-exome sequencing of fresh colon cancer tissue revealed a *KRAS* mutation. We consider that *KRAS* is abnormally regulated due to neurofibromin 1, which is unable to produce normal RAS protein, leading to intracellular signal transduction disorder, uncontrolled cell proliferation and cancer. Patients with NF1 have a high risk of developing malignant tumours, but there are currently no targeted drugs for the effective treatment of NF1. For patients with NF1, regular physical examination, early discovery, and early treatment are recommended.

In conclusion, we present a rare patient with NF1 associated with multiple GISTs and ascending colon adenocarcinoma. Patients with NF1 often have benign tumours and are more likely to develop malignant tumours than normal people. Therefore, these patients should undergo regular physical examinations so that early discovery and treatment can be achieved.

## Data Availability

Data related to this case report can be acquired from the corresponding author.

## Abbreviations

CA125: Carbohydrate antigen 125
CA19–9: Carbohydrate antigen 199
CA72–4: Carbohydrate antigen 199
CEA: Carcinoembryonic antigen
CT: Computed tomography
FOLFOX: Oxaliplatin + calcium folinate + fluorouracil
GIST: Gastrointestinal stromal tumour
IHC: Immunohistochemistry
NF1: Neurofibromatosis type 1
NGS: Next-generation sequencing
WES: Whole-exome sequencing
wt-p53: Wild-type p53
